# Disruption of Cholinergic Circuits as an Area for Targeted Drug Treatment of Alzheimer’s Disease: In Vivo Assessment of Short-Term Plasticity in Rat Brain

**DOI:** 10.3390/ph13100297

**Published:** 2020-10-09

**Authors:** Vergine Chavushyan, Ani Soghomonyan, Gohar Karapetyan, Karen Simonyan, Konstantin Yenkoyan

**Affiliations:** 1 Laboratory of Neuroscience, Yerevan State Medical University after M. Heratsi, Yerevan 0025, Armenia; verginechavushyan@gmail.com (V.C.); ani.soghomonyan@list.ru (A.S.); googakarapetyan@gmail.com (G.K.); 2 Laboratory of Neuroendocrine Relations, L. Orbeli Institute of Physiology of NAS, Yerevan 0028, Armenia; karensimonyan1986@yahoo.com; 3 Department of Biochemistry, Yerevan State Medical University after M. Heratsi, Yerevan 0025, Armenia

**Keywords:** amyloid-beta 25–35, nucleus basalis magnocellularis, basolateral amygdala, hippocampus, cholinergic circuit, short-term plasticity

## Abstract

The search for new therapeutics for the treatment of Alzheimer’s disease (AD) is still in progress. Aberrant pathways of synaptic transmission in basal forebrain cholinergic neural circuits are thought to be associated with the progression of AD. However, the effect of amyloid-beta (Aβ) on short-term plasticity (STP) of cholinergic circuits in the nucleus basalis magnocellularis (NBM) is largely unknown. STP assessment in rat brain cholinergic circuitry may indicate a new target for AD cholinergic therapeutics. Thus, we aimed to study in vivo electrophysiological patterns of synaptic activity in NBM-hippocampus and NBM-basolateral amygdala circuits associated with AD-like neurodegeneration. The extracellular single-unit recordings of responses from the hippocampal and basolateral amygdala neurons to high-frequency stimulation (HFS) of the NBM were performed after intracerebroventricular injection of Aβ 25–35. We found that after Aβ 25–35 exposure the number of hippocampal neurons exhibiting inhibitory responses to HFS of NBM is decreased. The reverse tendency was seen in the basolateral amygdala inhibitory neural populations, whereas the number of amygdala neurons with excitatory responses decreased. The low intensity of inhibitory and excitatory responses during HFS and post-stimulus period is probably due to the anomalous basal synaptic transmission and excitability of hippocampal and amygdala neurons. These functional changes were accompanied by structural alteration of hippocampal, amygdala, and NBM neurons. We have thus demonstrated that Aβ 25–35 induces STP disruption in NBM-hippocampus and NBM-basolateral amygdala circuits as manifested by unbalanced excitatory/inhibitory responses and their frequency. The results of this study may contribute to a better understanding of synaptic integrity. We believe that advancing our understanding of in vivo mechanisms of synaptic plasticity disruption in specific neural circuits could lead to effective drug searches for AD treatment.

## 1. Introduction

Central cholinergic network shortfalls are one of the most persistent neuropathological footprints in Alzheimer’s disease (AD) [1,2,3]. Recent in vivo investigations revealed that intrathecal injected or virally-delivered expression of amyloid-β (Aβ) peptide induces the reorganization of the cholinergic network within selected brain regions—the cerebral cortex, septohippocampal pathway, and nucleus basalis [4,5,6,7,8,9,10]. The high density of the innervation of cholinergic type is found in the hippocampus, entorhinal cortex, and amygdala [11,12,13]. The failure of cholinergic innervation in AD is more pronounced in the entorhinal cortex, related to a serious neurofibrillary degeneration and cell depletion in the basal nucleus complex [14,15]. The representative of the Meynert’s basal nucleus of primates in a rodent’s brain is the nucleus basalis magnocellularis (NBM). The NBM sends direct cholinergic projections to the basolateral amygdaloid nuclei [16]. In addition, even though the NBM does not transfer direct cholinergic projections to the hippocampus, NBM abrasion was found to weaken the basal synaptic responsiveness, short- and long-term neural plasticity in the rats’ hippocampus [17]. The NBM-hippocampus, and NBM-amygdala circuits can hold network fluctuations for years before the AD characteristic phenotype comes into play. On the other hand, one of the most sensitive “markers” to reveal these changes is the analysis of the short-term plasticity (STP) patterns. In vitro investigations have reported that STP has a deep influence on temporal filtering [18], network stability [18], and working memory [19]. Moreover, there is a bidirectional interplay between STP and long-term plasticity [20,21], and this interaction occurs on multiple timescales [22]. STP is critically important in development [23] and age-related disorders [24], including AD [25,26], but there is a lack of knowledge about its specific role, particularly in the context of local circuits, such as the cholinergic system affected in the AD brain.

We have already shown changes in the different forms of STP in the hippocampus during high-frequency stimulation (HFS) of the entorhinal cortex associated with AD-like pathology [27,28,29]. Moreover, our previous studies revealed a deficit of working and spatial memory in rats following modeling of AD by intracerebroventricular (i.c.v.) injection of Aβ 25–35, at later dates (60th, 90th days and more after Aβ 25–35 administration) [28,30,31].

We conducted a detailed in vivo study to assess the cascade of synaptic abnormalities in the hippocampus and basolateral amygdala during HFS of cholinergic NBM in an AD-like rat model induced by Aβ 25–35. We have demonstrated the aberrant excitatory/inhibitory (E/I) balance within the cholinergic network and impaired STP, reduced augmentation and/or depression during tetanization (HFS) and post-tetanization courses. The alterations in circuit activity allow cells to balance E/I and thereby prevent pathological states of hyper- or hypo-excitability of the network by homeostatic plasticity compensatory mechanism.

## 2. Results

**Figure 1:**
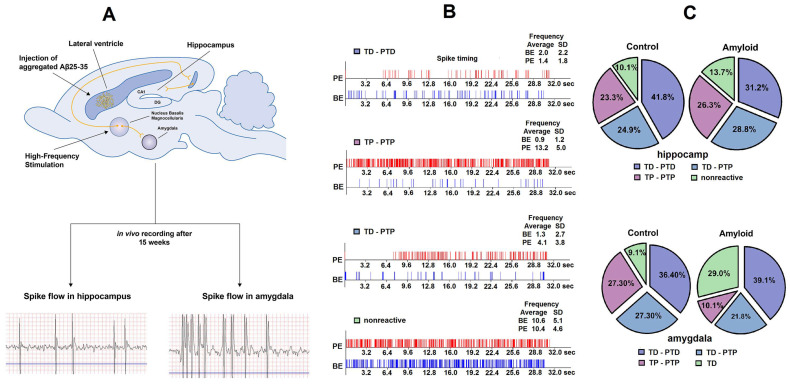
Graphical illustration of the whole electrophysiological experiment. (**A**) Typical example of in vivo recording of spike flow in single hippocampal and amygdala neurons. (**B**) Examples of spike activity of a single neuron: pulse flow in a real-time 30 s before stimulation (BE, before the event) and 30 s after stimulation (PE, post-event). (**C**) The percentage distribution of specific types of responses (tetanic depression-post-tetanic depression (TD-PTD), tetanic potentiation-post-tetanic potentiation (TP-PTP), tetanic depression-post-tetanic potentiation (TD-PTP), and no reactivity) of hippocampus and amygdala neurons to high-frequency stimulation of nucleus basalis magnocellularis in the control and amyloid groups.

**Figure 2:**
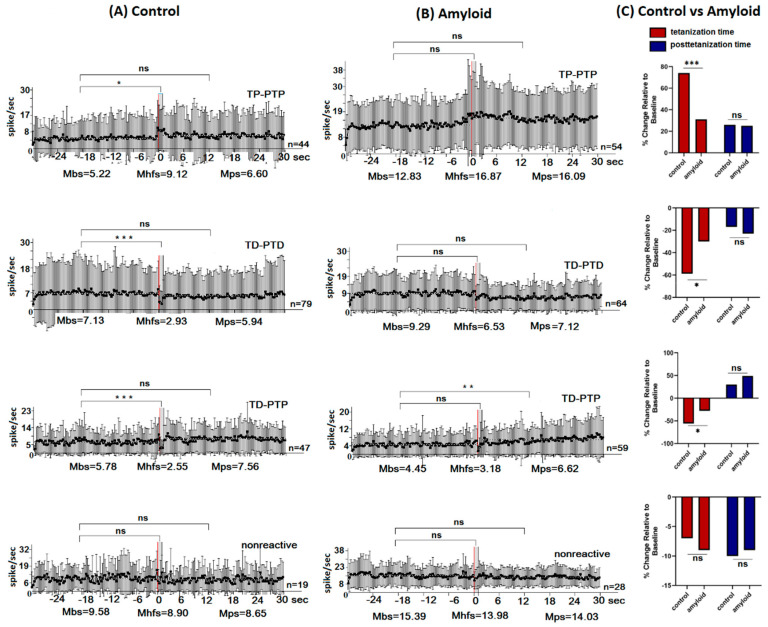
The average peri-stimulus time histogram of spike frequency in hippocampal neurons. (**A**,**B**). The mean peri-stimulus frequency diagrams built on the basis of pre-stimulus and post-stimulus manifestations of spike activity of single hippocampal neurons to high-frequency stimulation of nucleus basalis magnocellularis in a real-time 30 s before stimulation (Mbs), 30 s post-stimulation (Mps) and during high-frequency stimulation (Mhfs), exhibiting the specified types of responses (TP-PTP, TD-PTD, TD-PTP,) and nonreactivity in the control (**A**) and amyloid (**B**) groups. The data are presented as mean ± SD; *n* = number of neurons. The statistical significance of a difference from baseline (Mbs) was estimated according to the unpaired Student’s *t*-test, * *p* < 0.05, ** *p* < 0.01, and *** *p* < 0.001. (**C**). The bar diagrams show the average % changes relative to the baseline (Mbs, zero level) in responses of tetanization time (red) and post-tetanization time (blue) in control vs. amyloid groups in the population of neurons with a given type of response. Statistical significance was calculated using Fisher’s exact test, * *p* < 0.05.

**Figure 3:**
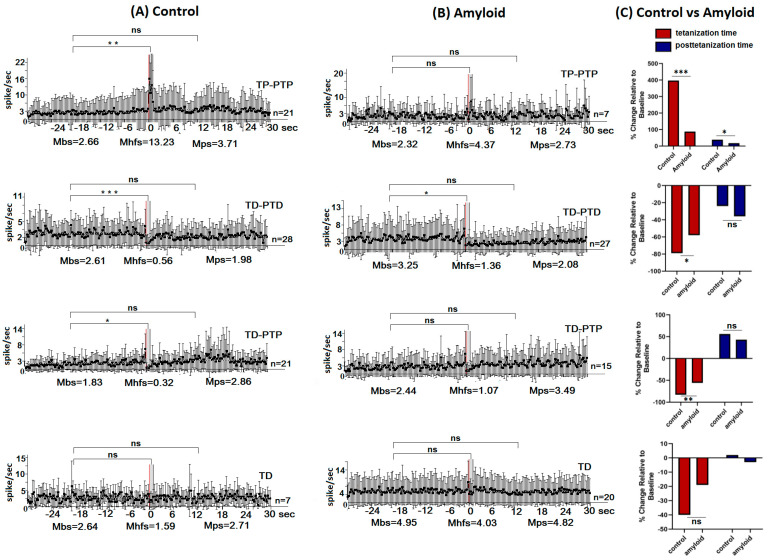
The average peri-stimulus time histogram of spike frequency in amygdale neurons. (**A**,**B**). The mean peri-stimulus frequency diagrams built on the basis of pre-stimulus and post-stimulus manifestations of spike activity of single amygdala neurons to high-frequency stimulation of nucleus basalis magnocellularis in a real-time 30 s before stimulation (Mbs), 30 s post-stimulation (Mps) and during high-frequency stimulation (Mhfs) exhibiting the specified type of responses (TP-PTP, TD-PTD, TD-PTP, TD) in the control (**A**) and amyloid (**B**) groups. The data are presented as mean ± SD; *n* = number of neurons. The statistical significance of a difference from baseline (Mbs) was estimated according to the unpaired Student’s *t*-test, * *p* < 0.05, ** *p* < 0.01, and *** *p* < 0.001 (**C**). The bar diagrams show the average % changes relative to baseline (Mbs, zero level) in responses of tetanization time (red) and post-tetanization time (blue) in the control vs. amyloid groups in the population of neurons with a given type of response. Statistical significance was calculated using Fisher’s exact test, * *p* < 0.05, ** *p* < 0.01, and *** *p* < 0.001.

Electrophysiological and morphological studies were carried out after 15 weeks of Aβ 25–35 and vehicle injections (Figure 1A). A total of 189 hippocampal and 77 amygdala neurons in the control group, 205 hippocampal, and 69 basolateral amygdala neurons in the amyloid group were recorded. The stimulation current amplitude was 0.10 mA for the control, and 0.12–0.14 mA for the amyloid group, which implies reduced excitability of tissues in the amyloid group. After recording the neuronal activity in animals of this group, all single-unit recordings were sorted according to the type of response. The analysis of spike frequency revealed an impulse flow acceleration during HFS (tetanic potentiation, TP) and post-stimulus period (post-tetanic potentiation, PTP), as well as an impulse flow deceleration during HFS (tetanic depression, TD) and post-stimulus period (post-tetanic depression, PTD) (Figure 1B). Different combinations of responses such as TD-PTD, TD-PTP, TP-PTP were recorded (Figure 1B). In each experimental group, the relative contribution of excitatory or inhibitory responses was calculated as a percentage share of the analyzed neurons with the corresponding type of response (Figure 1C). The mean/average frequency histograms for neurons with specific responses were built based on peri-stimulus spiking analysis for given populations (Figure 2 and Figure 3).

### 2.1. Excitatory and Inhibitory Responses of Hippocampal and Amygdala Neurons to HFS of NBM

The relative distribution of responses of hippocampal neurons to HFS of NBM in amyloid and control groups revealed a statistically significant decrease in TD-PTD (31.2% vs. 41.8%, *p* = 0.03). At the same time, the differences in TD-PTP (28.8% vs. 24.9%), TP-PTP (26.3% vs. 23.3%), and nonreactive responses (13.7% vs. 10.1%) in the amyloid and control groups were not significant (ns) (Figure 1C). The relative distribution of responses of amygdala neurons to HFS of NBM indicates a statistically significant decrease in TP-PTP responses (10.1% vs. 27.3%, *p* = 0.01) and a significant increase in TD (29% vs. 9.1%, *p* = 0.002) in the amyloid group (Figure 1C). At the same time, the difference in TD-PTD (39.1% vs. 36.4%) and TD-PTP responses (21.8% vs. 27.3%) between amyloid and control groups were not significant. (Figure 1C).

### 2.2. Real Time Pre- and Post-stimulus Spike Activity and Intensity of Responses of Hippocampal and Amygdala Neurons to HFS of NBM

We have evaluated the baseline/background activity level and the frequency of the excitatory and inhibitory responses of a single neuron of the hippocampus and amygdala to HFS of NBM. The baseline and HFS-induced responses were estimated in real-time in a population of neurons with similar responses, based on the mean levels of the spike activity frequencies before stimulation (Mbs)—baseline, post-stimulation (Mps) course, and in course of HFS (Mhfs). The intensity levels of TP, PTP, TD, and PTD were calculated as a percentage of an increase or decrease in spike frequency compared to baseline activity. Neurons in which an average spike frequency during stimulation (Mhfs) and post-stimulus time (Mps) differed from baseline (Mbs) by at least 10% according to statistical analysis were classified as nonresponsive units—nonreactive neurons.

The background/baseline (Mbs) spike train frequency of the amyloid group’s hippocampal neurons was significantly higher in the neural populations with TP-PTP (12.83 vs. 5.22 spike/s), TD-PTD (9.29 vs. 7.13 spike/s), and non-reactive (15.39 vs. 9.58 spike/s) (Figure 2B) rates compared to the control group (Figure 2A).

In the amyloid group (Figure 2B,C) of hippocampal neurons, the frequency rate of inhibitory responses increased and excitatory responses decreased compared to the control (Figure 2A,C). In the population of hippocampal neurons exhibiting TP-PTP pattern, the stimulation period frequency was 74.7% over the baseline (Mhfs = 9.12 spike/s; Mbs = 5.22 spike/s) in the control and 31.4% over the baseline (Mhfs = 16.87 spike/s; Mbs = 12.83 spike/s) in amyloid groups (Figure 2C, ns). The difference of PTP in the groups that are compared was insignificant. In the hippocampal neurons with TD-PTD response pattern, the stimulation period frequency was 59% below baseline (Mhfs = 2.93 spike/s; Mbs = 7.13 spike/s) in control and 29.7% (Mhfs = 6.53 spike/s; Mbs = 9.29 spike/s) in amyloid groups (Figure 2C, *p* = 0.03); while the difference in post-stimulation period was insignificant. The stimulation period frequency in the neurons with TD-PTP response was 55.9% below baseline activity (Mhfs = 2.55 spike/s; Mbs = 5.78 spike/s) in the control group and 28.5% (Mhfs = 3.18 spike/s; Mbs = 4.45 spike/s) in the amyloid group (Figure 2C, *p* = 0.04); the difference of PTP rate was insignificant. The difference in the population of neurons showing a non-reactive pattern was also not significant (Figure 2C).

The higher level of background (Mbs) activity in amygdala neurons was recorded in the amyloid group (Figure 3B) compared to the control group (Figure 3A) (3.25 vs. 2.61; 2.44 vs. 1.83; 4.95 vs. 2.64 spike/s), with the exception of a neuronal population showing a TP-PTP pattern.

The inhibitory and excitatory responses of amygdala neurons were found to be lower in the amyloid group (Figure 3B,C) compared to the control group (Figure 3A,C). So, in the population of amygdala neurons exhibiting TP-PTP the stimulation period frequency was 397% over baseline (Mhfs = 13.23 spike/s; Mbs = 2.66 spike/s) in the control and 88.4% (Mhfs = 4.37 spike/s; Mbs = 2.32 spike/s) in the amyloid groups (Figure 3C *p* = 0.0001); the difference of PTP response rate in compared groups was also significant (Figure 3C, *p* = 0.0001). In the amygdala neurons with TD-PTD tetanization time, depression levels were 78.5% below baseline (Mhfs = 0.56 spike/s; Mbs = 2.61 spike/s) in the control and 58.2% (Mhfs = 1.36 spike/s; Mbs = 3.25 spike/s) in the amyloid groups (Figure 3C, *p* = 0.02); the difference in intensity of PTD was not significant (Figure 3C). As regards tetanization time depression in the neurons with TD-PTP responses, tetanic depression levels were 82.5% below baseline activity (Mhfs = 0.32 spike/s; Mbs = 1.83 spike/s) in the control and 56.2% (Mhfs = 1.07spike/s; Mbs = 2.44 spike/s) in the amyloid groups (Figure 3C, *p* = 0.003); the difference of intensity of PTP was not significant (Figure 3C). The rate difference in the population of neurons showing only TD, was not significant (Figure 3C).

### 2.3. Morphological Study: Changes in Hippocampus, Amygdala and NBM

**Figure 4:**
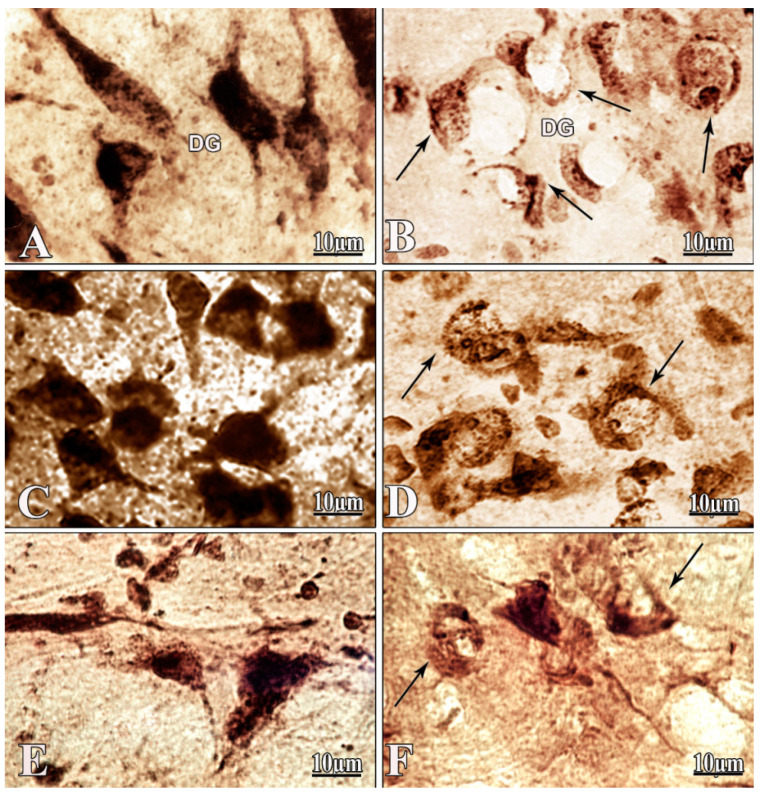
Images of morphologically examined neurons of hippocampus, amygdala, and nucleus basalis magnocellularis (NBM) in the control and amyloid groups. Frontal slices of hippocampal (**A**), amygdala (**C**), and NBM (**E**) neurons of the control rat brain. Degenerated, swollen neurons with eccentrically disposed nuclei (indicated by black arrows) of the hippocampus (**B**), amygdala (**D**), and NBM (**F**) after bilateral intracerebroventricular (i.c.v.) injection of Aβ 25–35. DG—dentate gyrus of the hippocampus. Magnification: ×1000 (**A**–**F**).

Results of the morphological study revealed a significant degeneration of hippocampal, amygdala, and NBM neurons after i.c.v. administration of Aβ 25–35 (Figure 4A–F). Interestingly, the most affected neurons were found in the hippocampus. In the swollen hippocampal neurons, central chromatolysisis was observed and the nuclei were localized eccentrically (Figure 4B). The morphological pattern in the amygdala showed resemblance to the acute swelling of neurons, which was a purely ordinary type of cellular pathology. These cells were swollen with an eccentrically located nucleus and the phenomenon of central chromatolysis was observed. In some areas, cells with sediment distributed in a thick layer under the cell membrane were observed (Figure 4D). The neurons of NBM were wrinkled, their shape and size were disrupted, the processes were not detected, and the eccentrically located nucleus was also revealed (Figure 4F).

## 3. Discussion

There is strong experimental support suggesting that hyperactivity of hippocampal as well as cortical neurons is one of the earliest dysfunctions initiated by Aβ [32,33]. Our current study shows that i.c.v. administration of Aβ 25–35 increases the background and post-stimulus spike frequency of hippocampal and amygdala neurons. This hyperactivity proves that Aβ exposure leads to the functional abnormality of the neurons possibly as a consequence of glutamatergic excitotoxicity. Abnormally increased Aβ blocks neuronal glutamate uptake at synapses, which enhances glutamate levels in the synaptic cleft [34]. Moreover, Aβ-induced synaptic collapse may be a result of a primary increase in synaptic activation by glutamate followed by synaptic NMDAR desensitization, NMDAR, and AMPAR internalization [34,35,36]

Alteration in the variables of presynaptic neurotransmitter vesicle stores and the Ca^2+^ microenvironment involves the strength and fidelity of PTP, and therefore interferes with long-term plasticity gating within individual synapses and across networks [37]. PTP, which is a form of presynaptic short-term plasticity (STP), results from a presynaptic increase of synaptic vesicle deliverance lasting for tens of seconds after discontinuance of tetanic stimulation [38]. Even though the peak magnitude of PTP initially depends on the enhancement in deliverance possibility caused by post-tetanic residual calcium [39], the elevation in the easily releasable pool size lasts longer than that in release probability [40]. In so far as enhanced production probability is positively associated with PTP expression [40], we can conclude that nonsignificant changes in PTP compared to baseline level most probably indicate the decreased release possibility and readily releasable pool size after Aβ 25–35 exposure.

The evidence reported in this study demonstrates that Aβ 25–35 intoxication stimulates anomalous synaptic activity in hippocampal and amygdala nerve cells, resulting in the imbalance of E/I responses evoked by HFS of NBM. Aβ 25–35 induced opposite changes in the E/I ratio. This is reflected by the prominent decline in the quantity of the hippocampal neurons displaying inhibitory responses (TD-PTD) and amygdala neurons with excitatory responses (TP-PTP) in the amyloid group. These shifts were less expressed in the hippocampal neurons compared to the amygdala, apparently due to the lack of direct projections from NBM to the hippocampus. These effects could apparently be due to the substantial structural damage of hippocampal, amygdala, and NBM neurons in the case of i.c.v. administration of Aβ 25–35.

It is shown that Aβ 25–35 affects the neuronal excitability in the cholinergic basal forebrain [8] and potentiates the Ca^2+^ influx through the L-type voltage-gated Ca^2+^ channels [41] in CA1 neurons of the hippocampus. In its turn, disruption of presynaptic Ca^2+^ channels results in the STP alterations and imbalances in hippocampal E/I circuits [42]. Modulation of Ca^2+^ channel levels plays a key role in tuning neurotransmitter release by calibrating synaptic function [36]. The effects of amyloid peptides might also be partially due to the interaction with the nicotinic acetylcholine receptors (nAChRs), thus altering their function [43,44]. Moreover, it was shown that in the hippocampus, nAChR activity can increase or weaken synaptic plasticity, depending on the type of neuron and timing of the nAChR activity [45]. Hence it could be speculated that the opposite effects of Aβ 25–35 in the hippocampus and amygdala are determined by the differential response of the corresponding nAChR receptors. The striking interplay in target-specific homeostasis modulates the efficacy of neurotransmission required for both hypo- and hyper-innervation to maintain stable synaptic strength [35]. Presynaptic plasticity optimally tunes presynaptic filtering, acting as a gain controller to amplify or depress transmission maximizing the efficiency of information transfer [46].

In addition to the described imbalance in E/I, we also evaluated the frequency of the excitatory and inhibitory responses to HFS. Aβ 25–35 decreases the intensity of tetanic potentiation and depression in hippocampal and amygdala neurons to HFS of cholinergic NBM, apparently due to the altered levels of neurotransmitters. Sustained presynaptic activation can result in deeper collapse that recovers more slowly [47], and therefore possibly PTD responses as observed in the current study are the consequences of the slow recovery following HFS. Although the molecular mechanisms responsible for the HFS-driven responses are still debated, one could speculate that, for instance, tetanic activation can increase synaptic strength for the period from only tens of seconds to minutes via the augmentation and PTP [47]. PTP can be explained by the temporary enhancement in synaptic strength lasting tens of seconds to minutes that follows the continuous HFS [48]. The total synaptic enhancement is a result of facilitation, PTP, and depression [38]. In the amyloid group an average magnitude of TD and TP decrease may reflect that postsynaptic mechanisms can also mediate STP, and this can make their characterization difficult. For instance, saturation of postsynaptic receptors can restrict responses, specifically when the possibility of the deliverance is elevated [49]. Postsynaptic receptors can be also desensitized, making them inaccessible for further activation, and causing the short-term drops in synaptic responses [50] and phase changes in neuronal postsynaptic spiking due to STP [51]. Our data on the decline in the equity ratio and expression of apparent excitatory responses in the amyloid group are consistent with data reported by Snyder et al. (2005) that Aβ lessens glutamatergic delivery through the regulation of NMDA receptor trafficking [52]. As the cholinergic and glutamatergic systems substantially interact during neurotransmission, variations in the glutamatergic signaling are connected with cholinergic splits found in AD [53], an aspect which supports the cholinergic hypothesis.

In general, our findings are consistent with the notion that both the expansive, diffuse innervations by the cholinergic system and the varied array of cholinergic activity apparently can edge the balance in favor of or against the induction of STP [45]. This type of synaptic activity enables the flexibility to locally adjust synaptic strength through input- and target-specificity to stabilize total network activity [35]. Therapeutic interventions aiming to stabilize synaptic integrity and cholinergic neurotransmission efficacy could have the potential to modify AD progression. 

We also want to note some limitations of this study. Among them are effects of amyloid-β 25–35 peptides due to differences in conformational structure and aggregation conditions in the internal in vivo environment [54]. The advantage of i.c.v. injection of Aβ 25–35 is that peptide penetrates into different brain structures nearby; however, it could limit the toxic dosage of amyloid in concrete AD target structures, e.g., hippocampus or basal ganglia. Using in vivo recording it is difficult to reveal the concrete type (e.g., interneuron, pyramidal neuron) and the number of neurons involved in every single recorded cellular response. Additionally, we did not identify what kind of specific neurons are responsible for each inhibitory or excitatory response—glutamatergic, GABAergic etc. In our further studies, using optogenetic tools, we should find the answers to the issues raised.

## 4. Methods and Materials

### 4.1. Animals

Experiments were performed on 20 Sprague-Dawley 12–14 month-old male rats. Animals with 220–280 g weight were kept under typical conditions of the laboratory vivarium. The experimental protocol satisfied the provisions of European Communities Council Directive (2010/63/UE) and was approved by the Ethics Committee of Yerevan State Medical University after Mkhitar Heratsi (N4 IRB APPROVAL, 15 November 2018).

### 4.2. Experimental Protocol

Animals were separated into two groups. Vehicle (double-distilled water)-treated animals were chosen as a control group. An i.c.v. injection of 3 µg/100 g body weight aggregated Aβ 25–35 was performed on the amyloid group.

### 4.3. Aβ 25–35 Peptide Preparation

Aβ 25–35 peptide was purchased from Sigma-Aldrich (St. Louis, MO, USA) and aggregated according to recommendations of the manufacturer [55]. In short, Aβ 25–35 was liquefied in sterile bi-distilled water at a concentration of 1 mg/mL, distributed into tubes and stored at −18 °C. “Aging” of the peptide was implemented by incubation at 37 °C for 4 days before surgery. Observation with light microscope established the existence of birefringent structures resembling fibrils, as well as globular aggregates.

### 4.4. Surgical Procedure

Animals were positioned in a stereotaxic frame and anesthetized with pentobarbital sodium solution (Nembutal 40 mg/kg) from Bio-Techne (Abingdon, UK). A midline sagittal scalp incision was then made. We used the following coordinates: AP −0.8–1 mm, L ±1.5 mm, DV +3.8 mm according to the stereotaxic atlas to drill holes over the lateral ventricles in the skull [56]. The injected solution was 3 µL of sterile double-distilled water (vehicle-treated) or 3 µL of aggregated Aβ 25–35 for the amyloid group. The injection was done into each cerebral lateral ventricle at a rate of 1 µL/min using the peristaltic pump. Syringe needles were kept in the injection sites for 3 min.

### 4.5. In Vivo Electrophysiology and Statistical Analysis

In vivo electrophysiological studies were carried out 15 weeks after the i.c.v. Aβ 25–35 injection. The rats were anesthetized with urethane (ethyl carbamate, 1.1 g/kg, i/p). Animals were immobilized with 1% dithylinum (25 mg/kg, i/p). Anesthetized and shaved rats were placed in a stereotactic frame and artificial ventilation was used. The stimulatory bipolar cylinder electrode was inserted following the stereotaxic coordinates [56] in the NBM (AP −1.08–1.1; L ±2.8–3; DV +7.4–7.8 mm). The glass recording electrode (tip diameter 1–2 μm, resistance, 1.5–2.5 MΩ) containing 2 M NaCl was repeatedly plunged into the ipsilateral dorsal hippocampus (CA1) and basolateral amygdala (the coordinates were AP −3.2–3.5; L ±1.5–3.5; DV +3.0–4.0 mm and AP −3.24; L ±5.4–5.8; DV +9.5–10.2 respectively) to record the spike activity flow of a single neuron. To ensure that coordinates were correct, we performed histology following an electrical injury at the end of the experiment. High frequency stimulation (HFS) (100 Hz during 1 s) was achieved using the rectangle charge for 0.05 ms with 0.10–0.14 mA amplitude. The control of the intensity of stimulation for an individual neuron was based on response threshold. Recording and analysis of the spike activity of single neurons were carried out by software [27,28] that selects spikes depending on amplitude and ignores artifacts of HFS, which allows estimation of the duration of interspike intervals (or spike frequency, spike/s) in a real-time within 30 s before stimulation (background pre-stimulus activity), 1 s of HFS (tetanization period activity) and 30 s after stimulation (post-stimulus activity). The statistical significance of the heterogeneity of interspike intervals (or spike frequency) of the pre- and post-stimulus impulse flow was analyzed by Student’s *t*-test and Mann–Whitney U test. The average peri-stimulus time histogram (PSTH) of neurons with uniform responses was constructed based on the analysis of peri-stimulus firing rate for given populations. Intra-population variance ratio between time intervals during HFS and post-stimulation compared to baseline was calculated using Student’s *t*-test. The statistical significance between control and amyloid groups was estimated according to chi-square and Fisher’s exact tests.

### 4.6. Morphological Study

Morphological and histochemical studies were carried out by the method of revealing of Ca^2+^-dependent acid phosphatase activity. The method is a variation of Nissl staining and Golgi silver impregnation [28,57]. Formalin fixation with 5% buffered neutral formalin for 24–48 h at 4 °C was done for the isolated brains. Then, 40–50 µm thick frontal free-flow frozen slices of hippocampus, amygdala, and NBM were cut, washed in distilled water, and taken into incubation mixture, which contained 0.4% lead acetate, 1 M acetate buffer (pH 5.6), 2% sodium glycerophosphate for 2–3 h at 37 °C. Then, the slices were washed in distilled water, put in 3% sodium sulfide solution, washed again in distilled water and embedded into Canada balsam.

## 5. Conclusions

The modern concept of synaptic neurotransmission and the cholinergic hypothesis revolutionized the field of AD research. Cholinesterase inhibitor-based therapy resulted in significant symptomatic improvement in patients with AD, thus validating the cholinergic system as an important therapeutic target in the disease. Our findings showed that Aβ peptides, the main triggers of AD, induce a disruption in the homeostatic nature of cholinergic circuits through disturbance of synaptic integrity and alterations of the STP. The results of this study may contribute to a better understanding of the involvement of STP in the pathophysiology and treatment strategies of AD. We suppose that our findings will give a new perspective on mechanisms to prioritize AD cholinergic therapeutics.
